# Supplementary material for: Clinically used broad-spectrum antibiotics compromise inflammatory monocyte-dependent antibacterial defense in the lung
Source: Nat Commun. 2024 Mar 30;15:2788. doi: 10.1038/s41467-024-47149-z (PMC10981692; doi:10.1038/s41467-024-47149-z)
Supplement: Supplementary file 1 — Supplementary information [file 41467_2024_47149_MOESM1_ESM.pdf]

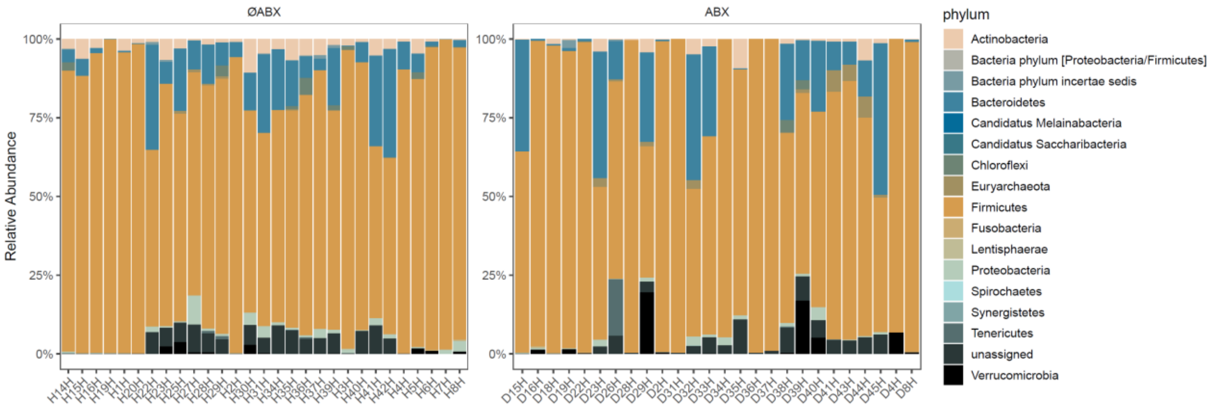

Suppl. Figure 1. Relative abundances of bacterial phyla in fecal samples of antibiotic-treated (ABX, n = 26) and untreated patients (ØABX, n=29).

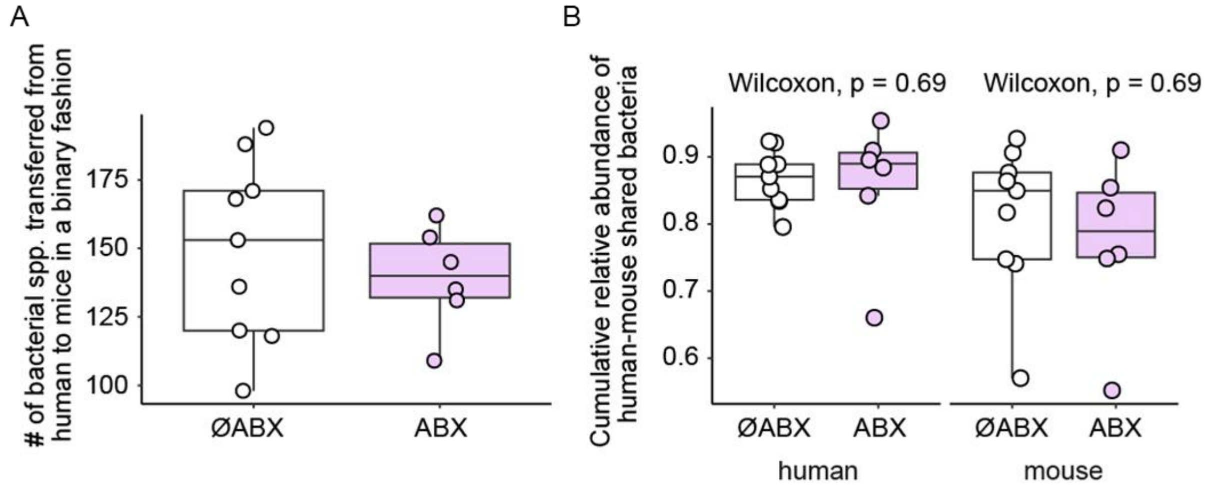

Suppl. Figure 2. Successful transfer of bacterial species from fecal samples of antibiotic-naïve patients (ØABX) or antibiotic-treated patients (ABX) into mice. (**A**) Number of bacterial species that were successfully transferred from patient fecal samples to mice. (**B**) Relative abundance of transferred bacterial species in the patient donor microbiota and in the mouse microbiota after transfer. Wilcoxon-Mann-Whitney *U* test (two-tailed) was used for relative abundance of transferred bacterial species. Each dot representing the data from one patient or mouse.

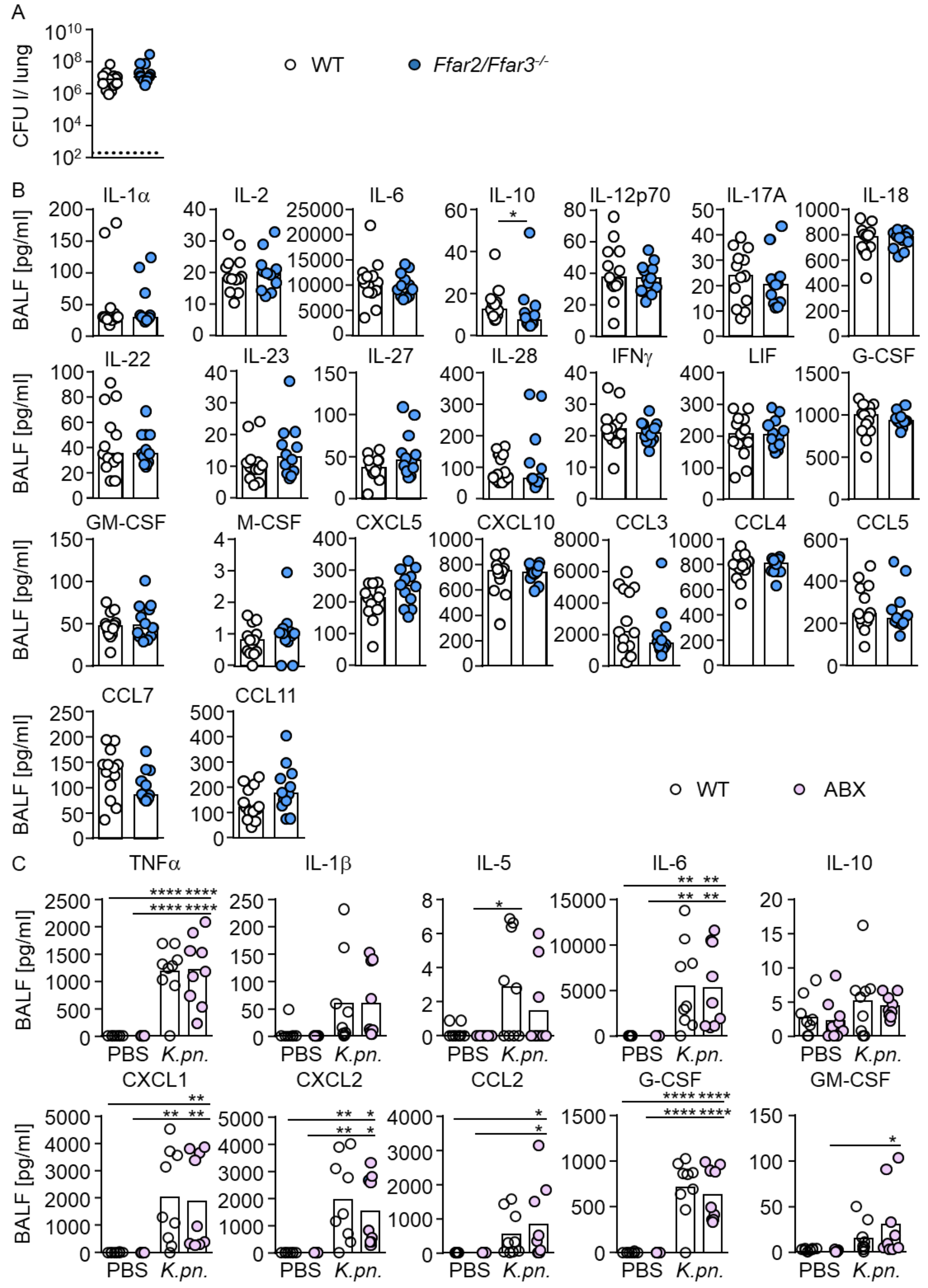

Suppl. Figure 3. The *K. pneumoniae*-induced inflammatory response seems not to be impaired in *Ffar2/Ffar3*$^{-/-}$ or microbiota-depleted mice. (**A-B**) Conventionally housed WT and *Ffar2/Ffar3*$^{-/-}$ mice were infected with *K. pneumoniae* for 12 h, bacterial loads (CFU) in lung tissues were counted (*n* = 18 for WT; *n* = 19 for *Ffar2/Ffar3*$^{-/-}$) (**A**) and cytokine levels in bronchoalveolar lavage fluid (BALF) were measured (*n* = 12 for WT; *n* = 13 for *Ffar2/Ffar3*$^{-/-}$) (**B**). (**C**) Conventionally colonized (CONV) and antibiotic-treated WT animals were infected

with *K. pneumoniae* or treated with PBS for 12 hours, and cytokine levels in BALF were assessed (n = 9 for CONV PBS, n = 9 for ABX PBS, n = 9 for CONV *K. pneumoniae* n = 9 for ABX *K. pneumoniae*). Mann-Whitney *U* test (two-tailed) was determined for bacterial loads (**A**) and inflammatory mediators' (**B**) from WT and *Ffar2/Ffar3*$^{-/-}$ mice. Kruskal-Wallis test followed by Dunn´s multiple comparison was applied to cytokine dataset from CONV and ABX mice (**C**). Values are shown as median (**A-C**), each dot represents the data from a single mouse. *$P < 0.05$, **$P < 0.01$, ***$P < 0.005$, *****$P < 0.001$.

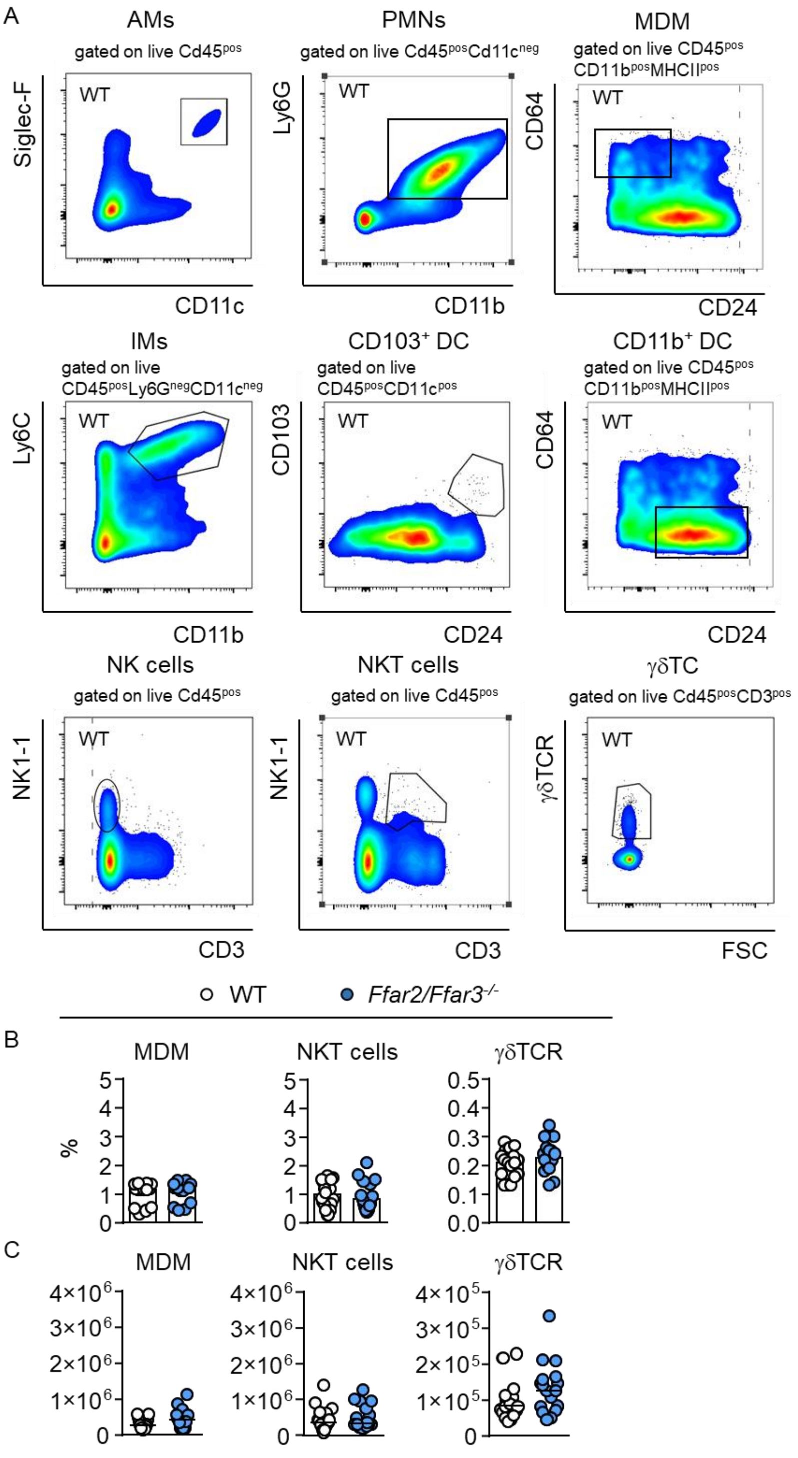

Suppl. Figure 4. Composition of lung immune cell populations in WT- and *Ffar2/Ffar3*$^{-/-}$ animals do not differ upon *K. pneumoniae* infection. (**A-C**) WT (n = 19) and *Ffar2/Ffar3*$^{-/-}$ (n = 18) mice were infected with *K. pneumoniae* for 12 hours. (**A**) FACS gating strategy of lung cells after exclusion of cell doublets and gating on live cells (CD45^{+}) for AMs, PMNs, eosinophils, IMs, CD11b^{+} DCs, CD103b^{+} DCs, monocyte derived macrophages (MDMs), NK

cells, natural killer T (NKT) cells and γδT cells (γδTC). (**B**-**C**) Percentage and numbers of MDMs, NKT and γδT cells were counted by FACS after staining of lung tissues. Mann-Whitney *U* test (two-tailed) was applied for lung cell populations (**B**-**C**). Values are shown as median, each dot represents the data from one mouse. $*P < 0.05$, $**P < 0.01$, $***P < 0.005$.

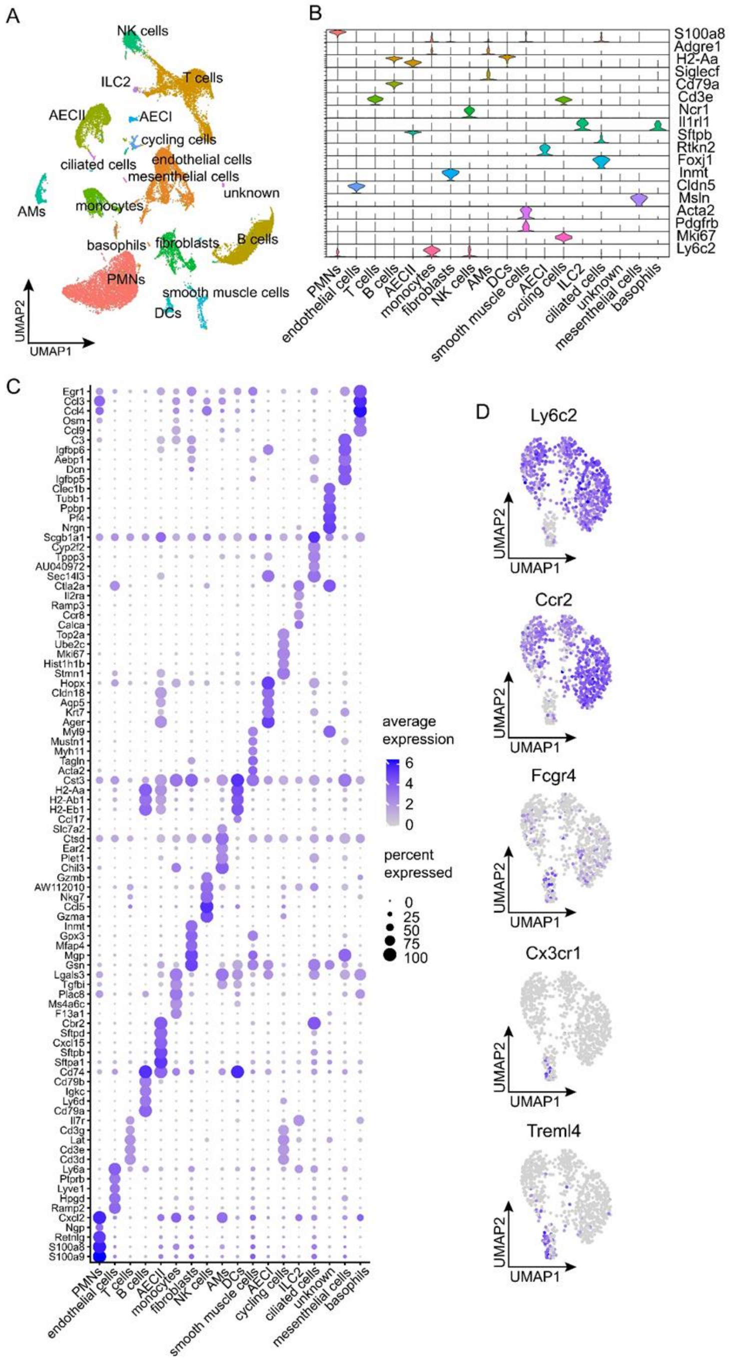

Suppl. Figure 5. scRNAseq of lung cells of WT and *Ffar2/Ffar3*$^{-/-}$ mice infected with *K. pneumoniae* for 12 h (*n* = 4 for WT PBS, *n* = 4 for WT infected, *n* = 4 for *Ffar2/Ffar3*$^{-/-}$ PBS, *n* = 4 for *Ffar2/Ffar3*$^{-/-}$ infected). (**A**) Two-dimensional embedding computed by UMAP on 30,215 computationally identified cells, (**B**) violin plots depicting representative marker genes for each cell type, and (**C**) dot plots of most differentially expressed genes for each cell population. (**D**) Normalized expression of marker genes of IMs and patrolling monocytes projected onto the UMAP embedding in Fig. 4G.

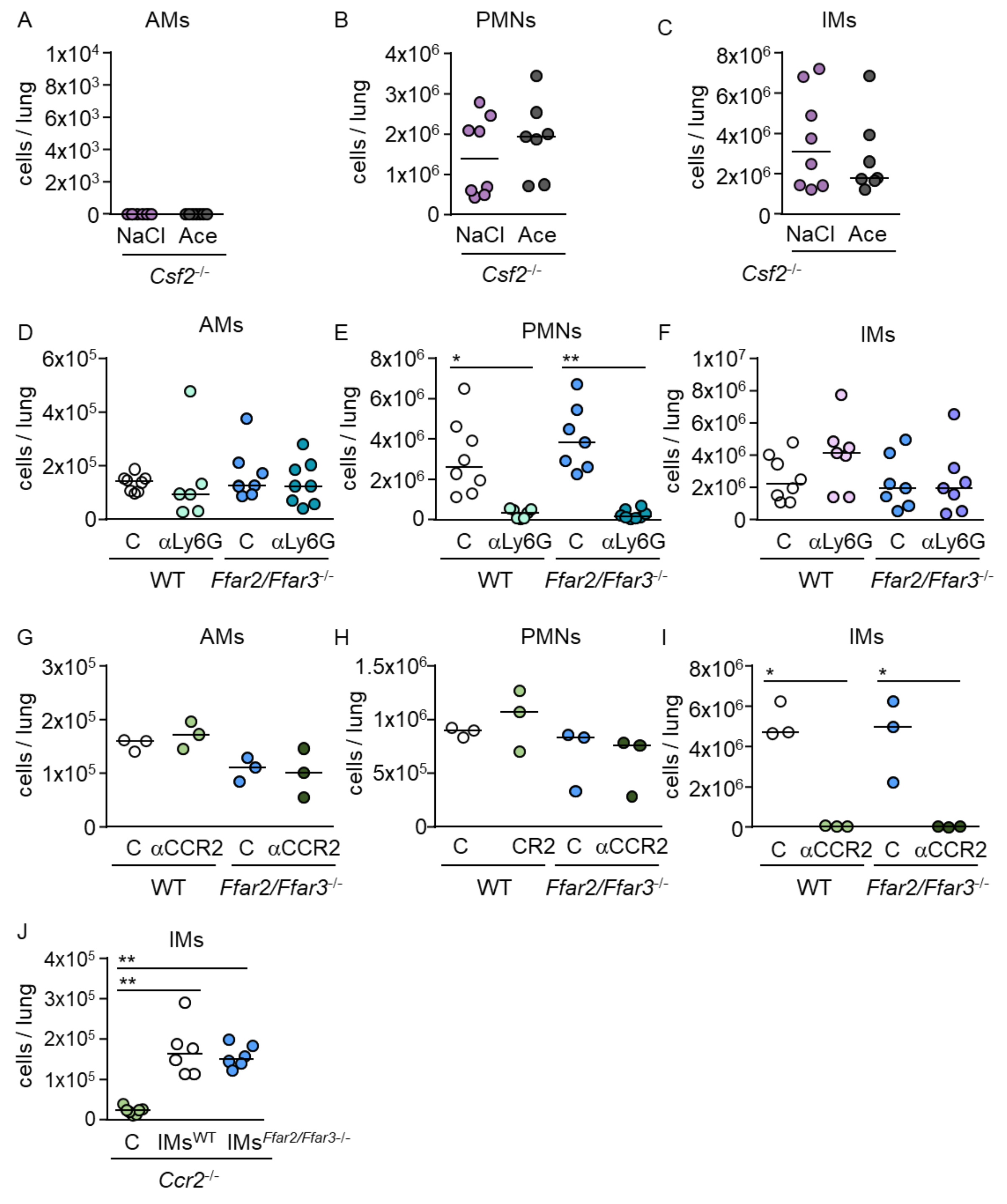

Suppl. Figure 6. Leukocyte numbers in lung tissues of *K. pneumoniae*-infected *Csf2*$^{-/-}$, WT and *Ffar2/Ffar3*$^{-/-}$ mice. (**A-C**) Numbers of AMs, PMNs, and IMs in *Csf2*$^{-/-}$ mice (NaCl; *n* = 8; *n* = 7 for acetate). (**D-F**) Numbers of AMs, PMNs, and IMs in WT and *Ffar2/Ffar3*$^{-/-}$ mice treated with control antibodies (C) or αLy6G (n = 8 for WT C, *n* = 7 for *Ffar2/Ffar3*$^{-/-}$ C, n = 7 for WT αLy6G; *n* = 8 for *Ffar2/Ffar3*$^{-/-}$ αLy6G). (**G-I**) Numbers of AMs, PMNs, and IMs in WT and *Ffar2/Ffar3*$^{-/-}$ mice treated with control antibodies (C) or αCCR2 (*n* = 3 for WT C, *n* = 3 for

Ffar2/Ffar3$^{-/-}$ C, n = 3 for WT αCCR2 ; *n* = 3 for *Ffar2/Ffar3*$^{-/-}$ αCCR2). **(J)** Number of IMs in *K. pneumoniae*-infected *Ccr2*$^{-/-}$ mice treated intravenously with PBS (C) or transplanted with IMs of WT or *Ffar2/Ffar3*$^{-/-}$ animals (n = 7 for PBS , *n* = 6 for WT αCCR2; *n* = 6 for *Ffar2/Ffar3*$^{-/-}$). Values are shown as median, each dot represents the data from a single mouse. *$P < 0.05$, **$P < 0.01$, ***$P < 0.005$.
