# Supplementary material for: Clinically used broad-spectrum antibiotics compromise inflammatory monocyte-dependent antibacterial defense in the lung
Source: Nat Commun. 2024 Mar 30;15:2788. doi: 10.1038/s41467-024-47149-z (PMC10981692; doi:10.1038/s41467-024-47149-z)
Supplement: Supplementary file 3 — Description of Additional Supplementary Files [file 41467_2024_47149_MOESM3_ESM.pdf]

<u>Description of Additional Supplementary Files</u>

Supplementary Data 1: Metadata of patients included in our study

Supplementary Data 2: SCFA KEGG KO module bins
