# Supplementary material for: Clinically used broad-spectrum antibiotics compromise inflammatory monocyte-dependent antibacterial defense in the lung
Source: Nat Commun. 2024 Mar 30;15:2788. doi: 10.1038/s41467-024-47149-z (PMC10981692; doi:10.1038/s41467-024-47149-z)
Supplement: Supplementary file 6 — Reporting Summary [file 41467_2024_47149_MOESM6_ESM.pdf]

nature portfolio

Corresponding author(s): Patrick J. Dörner, Harithaa Anandakumar, Ivo Röwekamp, Facundo Fiocca Vernengo, Belén Millet Pascual-Leone, Marta Krzanowski, Josua Sellmaier, Ulrike Brüning, Raphaela Fritsche, Lennart Pfannkuch, Florian Kurth, Miha Milek, Vanessa Igbokwe, Ulrike Löber, Birgitt Gutbier, Markus Holstein, Gitta Heinz, Mir-Farzin Mashreghi, Leon N. Schulte, Ann-Brit Klatt, Sandra Caesar, Sandra-Maria Wienhold, Stefan Offermanns, Matthias Mack, Martin Witzenrath, Stefan Jordan, Dieter Beule, Jennifer A. Kirwan, Sofia K. Forslund, Nicola Wilck, Hendrik Bartolomaeus, Markus M. Heimesaat, Bastian Opitz

Last updated by author(s): 19.02.2024

# Reporting Summary

Nature Portfolio wishes to improve the reproducibility of the work that we publish. This form provides structure for consistency and transparency in reporting. For further information on Nature Portfolio policies, see our Editorial Policies and the Editorial Policy Checklist.

## Statistics

For all statistical analyses, confirm that the following items are present in the figure legend, table legend, main text, or Methods section.

| n/a | Confirmed | |
|---|---|---|
| ☐ | ☒ | The exact sample size (*n*) for each experimental group/condition, given as a discrete number and unit of measurement |
| ☐ | ☒ | A statement on whether measurements were taken from distinct samples or whether the same sample was measured repeatedly |
| ☐ | ☒ | The statistical test(s) used AND whether they are one- or two-sided<br>*Only common tests should be described solely by name; describe more complex techniques in the Methods section.* |
| ☐ | ☒ | A description of all covariates tested |
| ☐ | ☒ | A description of any assumptions or corrections, such as tests of normality and adjustment for multiple comparisons |
| ☐ | ☒ | A full description of the statistical parameters including central tendency (e.g. means) or other basic estimates (e.g. regression coefficient) AND variation (e.g. standard deviation) or associated estimates of uncertainty (e.g. confidence intervals) |
| ☐ | ☒ | For null hypothesis testing, the test statistic (e.g. *F*, *t*, *r*) with confidence intervals, effect sizes, degrees of freedom and *P* value noted<br>*Give P values as exact values whenever suitable.* |
| ☒ | ☐ | For Bayesian analysis, information on the choice of priors and Markov chain Monte Carlo settings |
| ☒ | ☐ | For hierarchical and complex designs, identification of the appropriate level for tests and full reporting of outcomes |
| ☒ | ☐ | Estimates of effect sizes (e.g. Cohen's *d*, Pearson's *r*), indicating how they were calculated |

*Our web collection on statistics for biologists contains articles on many of the points above.*

## Software and code

Policy information about availability of computer code

Data collection

Data availability: Source data are provided with this paper. The raw microbiome shotgun sequencing and single-cell RNA sequencing data supporting the conclusions of this article are available under the BioProject ID PRJNA1064467 from the NIH Sequence Read Archive (https://www.ncbi.nlm.nih.gov/bioproject/PRJNA1064467).
Sequences retrieved from metagenomic shotgun sequencing were processed with the NGless pipeline (v1.3) 43. Reads were trimmed with a Phred-score <25 and discarded if the sequence length was below 45 bp. Contamination filtering was performed by mapping the reads against GRCh38.p14 for human-derived stool samples and with GRCm39 for mouse-derived stool samples, respectively. Both references have been masked by mapping bacterial genes from the proGenomes2 database 44. Taxonomic profiling was conducted using the NGless mOTUs module (v2.6). Functional profiling was performed by mapping reads to the Global Microbial Gene Catalog (GMGC, human-gut (v1)) 45 and further aggregated at the KEGG KO level. Samples with fewer than 1000 reads were excluded from the analysis. Species detected in at least 10% of the samples with an overall study-wide abundance of at least 10e-4 were retained for further analysis. Species level alpha and beta diversity were calculated using vegan (v2.6-4) package (Shannon index) and the stats (v4.2.2) package. The SCFA-pathways (acetate, butyrate,

| | |
|---|---|
| | propionate) were binned manually from KEGG KOs (see supplemental material 2). Orddom (v3.1) package was used to calculate Cliff's delta, as a measure of standardized effect sizes, of microbial species abundance and SCFA-pathway abundances across the different group comparisons<br><br>Code availability: The R code used for statistical analysis and to generate the figures in this study is archived in Zenodo with the DOI: 10.5281/zenodo.10526874. |
| Data analysis | Flow Jo V10, R (version 4.2.1) , statistical anaylsis was performed by using GraphPad Prism Version 9, Seurat package 46 (version 4.2.0)was used to integrate datasets and Volcano plots were generated using ggplot2 (v3.4.0) (Single-cell sequencing) |

For manuscripts utilizing custom algorithms or software that are central to the research but not yet described in published literature, software must be made available to editors and reviewers. We strongly encourage code deposition in a community repository (e.g. GitHub). See the Nature Portfolio guidelines for submitting code & software for further information.

## Data

Policy information about availability of data

All manuscripts must include a data availability statement. This statement should provide the following information, where applicable:

- Accession codes, unique identifiers, or web links for publicly available datasets
- A description of any restrictions on data availability
- For clinical datasets or third party data, please ensure that the statement adheres to our policy

All major data generated or analysed in this study are included in the article or supplementary information files. All other data relating to this study are available from the corresponding author on reasonable request.
Data availability: Source data are provided with this paper. The raw microbiome shotgun sequencing and single-cell RNA sequencing data supporting the conclusions of this article are available under the BioProject ID PRJNA1064467 from the NIH Sequence Read Archive (https://www.ncbi.nlm.nih.gov/bioproject/PRJNA1064467)

Code availability: The R code used for statistical analysis and to generate the figures in this study is archived in Zenodo with the DOI: 10.5281/zenodo.10526874.

## Research involving human participants, their data, or biological material

Policy information about studies with human participants or human data. See also policy information about sex, gender (identity/presentation), and sexual orientation and race, ethnicity and racism.

| | |
|---|---|
| Reporting on sex and gender | Sex and gender of patients were not considered relevant for this study |
| Reporting on race, ethnicity, or other socially relevant groupings | Race, ethinicity or other socially groupings were not considered relevant for this study |
| Population characteristics | The cohort of patients were selected randomly irrespective of age and sex, inclusion criteria were age ≥18 years, at least two days of therapy with broad-spectrum antibiotics (with activity against Gram-positive, Gram-negative, and anaerobic bacteria), or no antibiotic use for at least two months. Exclusion criteria included acute colitis and inflammatory bowel disease. |
| Recruitment | The cohort of patients was recruited between 2020 and 2021 in respiratory medicine wards at Charité. Patients gave their informed consent for sampling. Inclusion criteria were age ≥18 years, at least two days of therapy with broad-spectrum antibiotics (with activity against Gram-positive, Gram-negative, and anaerobic bacteria), or no antibiotic use for at least two months. |
| Ethics oversight | The human part of the study was approved by the institutional review board (Ethics Committee of the Charité Universitätsmedizin Berlin, identifier: EA4/232/19) and was registered at the clinical study registry of the Charité Universitätsmedizin Berlin (https://studienregister.charite.de, identifier: 3000023). Written informed consent was received from all participants. |

Note that full information on the approval of the study protocol must also be provided in the manuscript.

# Field-specific reporting

Please select the one below that is the best fit for your research. If you are not sure, read the appropriate sections before making your selection.

☒ Life sciences ☐ Behavioural & social sciences ☐ Ecological, evolutionary & environmental sciences

For a reference copy of the document with all sections, see nature.com/documents/nr-reporting-summary-flat.pdf

# Life sciences study design

All studies must disclose on these points even when the disclosure is negative.

| | |
|---|---|
| Sample size | sample sizes of in vivo and in vitro experiments were chosen to obtain significant results and prospective power analysis was performed with appropriate statistical methods. |
| Data exclusions | No data points were excluded |
| Replication | All major experiments in this manuscript were repeated at least three times successfully. |
| Randomization | The cohorts of patients were recruited between 2020 and 2021 at the clinic for Respiratory Medicine and Infectious Diseases at Charité. The selection of patients followed a cross-sectional approach. Group allocation was not random but according to prior/current treatment (at least two days of treatment with broad-spectrum antibiotics vs. no antibiotics use within at least two months prior). Patients gave their informed consent for sampling. Inclusion criteria were age ≥ 18 and the aforementioned treatment regimes. Exclusion criteria included acute colitis and inflammatory bowel disease.<br>Self-selection bias/other biases: Since patients were actively approached to be included in the study, selection bias might apply. Randomization and blinding were not feasible in this cross-sectional setting. Efforts were made to match patient characteristics in both groups, where possible.<br><br>Population characteristics: Covariate distributions (incl. age and sex) were similar in both groups and statistical differences were only found for serum levels of CRP and grounds for hospitalization (see Table 1). Since one group included patients receiving antibiotic therapy, these differences were expected.<br><br>Randomization: Group allocation in the patient cohorts was not random but according to prior/current treatment (control: no antibiotics use within at least two months vs. at least two days of treatment with broad-spectrum antibiotics). During patient enrollment efforts were made to match the covariate distribution across both cohorts. Due to the constraints imposed by the limited number of patients who had not received antibiotics for a minimum of two months (control group), patients from the antibiotic treatment group were matched onto the control group. |
| Blinding | During group allocation of the patient cohorts, blinding was not feasible. Sample processing and analysis were carried out by an external provider who was blinded for group allocation. |

# Reporting for specific materials, systems and methods

We require information from authors about some types of materials, experimental systems and methods used in many studies. Here, indicate whether each material, system or method listed is relevant to your study. If you are not sure if a list item applies to your research, read the appropriate section before selecting a response.

## Materials & experimental systems

| n/a | Involved in the study | |
|---|---|---|
| ☐ | ☒ | Antibodies |
| ☒ | ☐ | Eukaryotic cell lines |
| ☒ | ☐ | Palaeontology and archaeology |
| ☐ | ☒ | Animals and other organisms |
| ☐ | ☒ | Clinical data |
| ☒ | ☐ | Dual use research of concern |
| ☒ | ☐ | Plants |

## Methods

| n/a | Involved in the study | |
|---|---|---|
| ☒ | ☐ | ChIP-seq |
| ☐ | ☒ | Flow cytometry |
| ☒ | ☐ | MRI-based neuroimaging |

## Antibodies

| | |
|---|---|
| Antibodies used | CD11b-BV421 (Clone:M1/70,Cat #101251, Lot: B360436, BioLegend), Ly6G-BV510 (Clone: 1A8,Cat #127633, Lot B357022 BioLegend);CD45-FITC (Clone:30-F11,Cat #103107, Lot B350443 BioLegend), Ly6C-PerCP-Cy5.5 (Clone HK1.4, Cat #128028 Lot B358852 BioLegend), Siglec-F-PE (Clone E50-2440, Cat #552126, Lot 2151784 Becton Dickinson), CD11c-APC (clone N418, Cat # 117310, Lot B372999 BioLegend), CD4-BV510 (Clone RM4-5,Cat# 100553, Lot B370264 BioLegend),<br>δTCR-FITC (clone GL3, Cat #5536177, Lot 0000067419 Becton Dickinson), CD45-PerCP-Cy5.5 (clone 30-F11, Cat #557235 Lot 7125627 Becton Dickinson),<br>CD3-PE (clone 17A2, Cat #100206, Lot B166002 BioLegend), NK1.1-PECy7 (clone PK136, Cat # 108714 , Lot B348261 BioLegend),<br>CD24-BV450 (clone M1/69, Cat #101816, Lot B151476 BioLegend);CD11b-BV510 (Clone M1/70, Cat # 101245, Lot B313403 BioLegend), CD11c-FITC (clone N418, Cat # 117306, Lot B179353 BioLegend),<br>CD64-PE (clone X54-5/7.1, Cat # 139304, Lot B171679 BioLegend),<br>MHCII-PECy7 (clone M5/114.15.2, Cat # 107629, Lot B352997 BioLegend)<br>CD103-APC (clone M290, Cat # 562772, Lot 5262863 Becton Dickinson)<br>CD11b-PECy7 (clone M1/70, Cat #101216, Lot B360932 BioLegend), Ly6G-PE (clone 1A8, Cat #127607, Lot B371498 BioLegend), Ly6C-PerCP (clone HK1.4, Cat # 128028, Lot B358852 BioLegend), NK1.1-APC (clone S17016D, Cat # 156505, Lot B360021 BioLegend)<br>anti-Ly6G (clone 1A8; cat #BP0075-1, Lot 854522J3 BioXCell), anti-rat kappa immunoglobulin light chain (clone MAR 18.5;cat |

| | |
|---|---|
| | #BE0122, Lot 805321D1 BioXCell), aisotype control rat IgG2a (clone 2A3; cat # BP0089, Lot 815022F1 BioXCell) hashtag 1 (clone M1/42, cat #155801, Lot B312578, BioLegend ), hashtag antibody 2 (clone M1/42, cat #155803, lot B326543 BioLegend), hashtag 3 (clone M1/42, Cat #155805, Lot B322111, BioLegend), hashtag 4 (clone M1/42, Cat #155807, Lot B334831 BioLegend). rat anti-mouse CCR2 (clone MC-21, Lot 1480102, gifted by Matthias Mack) , isotype control IgG2b (clone RTK4530; Cat #400666, Lot B253170 BioLegend) |
| Validation | all antibodies are validated by the respective companies ( Becton Dickinson, BioLegend, BioXCell & DOI: 10.4049/ jimmunol.179.5.3099) |

# Animals and other research organisms

Policy information about studies involving animals; ARRIVE guidelines recommended for reporting animal research, and Sex and Gender in Research

| | |
|---|---|
| Laboratory animals | 8 to 16 weeks-old wild type C57Bl/6J, FFAR2/3-/-, CSF2-/- and CCR2-/- mice were used. Mice were bred under specific pathogen-free conditions under 22 °C, 50–55% relative humidity, and 12 h/12 h light/dark cycle conditions and were fed with rat/mouse maintenance food V1534-000 (Ssniff) |
| Wild animals | This study did not involve wild animals |
| Reporting on sex | Sex was not considered in this study. Mice were chosen by fitting age |
| Field-collected samples | This study does not involve field-collected samples |
| Ethics oversight | All animal experiments were carried out in strict adherence to the German law (Tierschutzgesetz, TierSchG), following the approval by the responsible institutional (Charité Universitätsmedizin Berlin) and governmental animal welfare authorities (LAGeSo Berlin, approval ID G0284/16 and G0010/22). |

Note that full information on the approval of the study protocol must also be provided in the manuscript.

# Clinical data

Policy information about clinical studies

All manuscripts should comply with the ICMJE guidelines for publication of clinical research and a completed CONSORT checklist must be included with all submissions.

| | |
|---|---|
| Clinical trial registration | The human part of the OBSERVATIONAL STUDY was approved by the institutional review board (Ethics Committee of the Charité Universitätsmedizin Berlin, identifier: EA4/232/19) and was registered at the clinical study registry of the Charité Universitätsmedizin Berlin (https://studienregister.charite.de, identifier: 3000023). |
| Study protocol | Inclusion criteria were age ≥18 years, at least two days of therapy with broad-spectrum antibiotics (with activity against Gram-positive, Gram-negative, and anaerobic bacteria), or no antibiotic use for at least two months. Exclusion criteria included acute colitis and inflammatory bowel disease. |
| Data collection | The cohort of patients was recruited between 2020 and 2021 in respiratory medicine wards at Charité. Patients gave their informed consent for sampling. Inclusion criteria were age ≥18 years, at least two days of therapy with broad-spectrum antibiotics (with activity against Gram-positive, Gram-negative, and anaerobic bacteria), or no antibiotic use for at least two months. Exclusion criteria included acute colitis and inflammatory bowel disease. Sex of participants are indicated but not further considered in the study design as we describe a general mechanism that is likely not to be sex/gender-specific. Compensation was not paid. |
| Outcomes | No clinical outcomes were defined. |

# Flow Cytometry

## Plots

Confirm that:

☒ The axis labels state the marker and fluorochrome used (e.g. CD4-FITC).

☒ The axis scales are clearly visible. Include numbers along axes only for bottom left plot of group (a 'group' is an analysis of identical markers).

☒ All plots are contour plots with outliers or pseudocolor plots.

☐ A numerical value for number of cells or percentage (with statistics) is provided.

## Methodology

| | |
|---|---|
| Sample preparation | Mice were sacrificed, hind limbs were separated and cleaned from surrounding tissues and dipped in ice-cold ethanol. Femur and tibia were disconnected, and bone marrow was collected by cutting off the ends and by flushing the bones with 10 mL sterile phosphate-buffered saline. Isolated bone marrow was filtered with a 70 µM cell strainer, spun down at 500 g for 10 min at 4°C, and red blood lysis was performed.<br>For Lung cells after euthanizing, broncho-alveolar lavage fluid (BALF) with cold PBS was performed; whole lungs were |

harvested afterwards, homogenized in sterile PBS and filtered through a 70 µM cell strainer.Homogenized lungs were incubated in DNAse/Collagenase at 37°C for 40 minutes followed by filtering of cell suspensions through a 70 µM cell strainer and red blood cell lysis to obtain a single cell suspension

| | |
|---|---|
| Instrument | FACSCanto II analyzer (Becton Dickinson), FACS AriaTM II SORP flow cytometer cell sorter (Becton Dickinson). |
| Software | FlowJo Version 10 |
| Cell population abundance | Lung cell suspensions contained approximately 85-90% intact immune cells, which were seperated during measurement from erythrocytes and cell depris by SSC/FSC gating. Gated bone marrow monocytes had an abundance of 6-9% in the bone marrow isolates which were seperated during measurement from erythrocytes and cell depris by SSC/FSC gating and had a purity above 95% |
| Gating strategy | lung cells were stained with LIVE/DEAD Fixable Cell Dead stain (Thermo Fisher Scientific) and with different combinations of the following anti-mouse antibodies: CD11b-BV421 (M1/70, BioLegend), Ly6G-BV510 (1A8, BioLegend), CD45-FITC (30-F11, BioLegend), Ly6C-PerCP-Cy5.5 (HK1.4, BioLegend), Siglec-F-PE (E50-2440, Becton Dickinson), CD11c-APC-N418, BioLegend), CD4-BV510 (RM4-5, BioLegend), γδTCR-FITC (GL3, Becton Dickinson), CD45-PerCP-Cy5.5 (30-F11, Becton Dickinson), CD3-PE (17A2, Becton Dickinson), NK1.1-PECy7 (PK136, BioLegend), CD24-BV450 (M1/69, Becton Dickinson), CD11b-BV510 (M1/70, BioLegend), CD11c-FITC (N418, BioLegend), CD64-PE (X54-5/7.1, BioLegend), MHCII-PECy7 (M5/114.15.2, BioLegend) and CD103-APC (M290, Becton Dickinson).Lung cell suspensions contained approximately 85-90% intact immune cells, which were seperated during measurement from erythrocytes and cell depris by SSC/FSC gating. Sorted IMs were defined as Ly6G-NK1.1-CD11b+Ly6Chi cells . Gated bone marrow monocytes had an abundance of 6-9% in the bone marrow isolates which were seperated during measurement from erythrocytes and cell depris by SSC/FSC gating and had a purity above 95% |

☒ Tick this box to confirm that a figure exemplifying the gating strategy is provided in the Supplementary Information.
